# Online Acceptance and Commitment Therapy and Nutrition Workshop for Parents of Children with Inflammatory Bowel Disease: Feasibility, Acceptability, and Initial Effectiveness

**DOI:** 10.3390/children8050396

**Published:** 2021-05-14

**Authors:** Sara Ahola Kohut, Inez Martincevic, Sheri L. Turrell, Peter C. Church, Thomas D. Walters, Natalie Weiser, Armanda Iuliano

**Affiliations:** 1 Department of Gastroenterology, Hepatology and Nutrition, The Hospital for Sick Children, Toronto, ON M5G 1X8, Canada; peter.church@sickkids.ca (P.C.C.); thomas.walters@sickkids.ca (T.D.W.); 2 Child Health Evaluative Sciences, SickKids Research Institute, Toronto, ON M5G 1X8, Canada; natalie.weiser@sickkids.ca (N.W.); armanda.iuliano@sickkids.ca (A.I.); 3 Department of Psychiatry, University of Toronto, Toronto, ON M5G 1X8, Canada; 4 Department of Clinical Dietetics, The Hospital for Sick Children, Toronto, ON M5G 1X8, Canada; inez.martincevic@sickkids.ca; 5 Life in Balance Therapy, Toronto, ON M6R 2L2, Canada; drsturrell@rogers.com; 6 Department of Paediatrics, University of Toronto, Toronto, ON M5G 1X8, Canada

**Keywords:** acceptance and commitment therapy, inflammatory bowel disease, parent intervention, ehealth, nutrition, mindfulness, interdisciplinary

## Abstract

Parents of children with inflammatory bowel disease (IBD) are important members of their healthcare team and influence their child’s adaptation to disease. The primary aim of this research was to test the feasibility and acceptability of a three-session online parent workshop based on acceptance and commitment therapy (ACT) and address concerns about eating well and nutrition in IBD. The secondary aim was to explore the initial effectiveness of this workshop in parent reported psychological flexibility, mindfulness, experiential avoidance, cognitive fusion, valued living, and symptoms of depression, anxiety, and stress. We used a single arm pragmatic prospective study design with parents of children attending the IBD program at a tertiary pediatric healthcare centre in Canada. Mixed methods patient reported outcomes were measured at baseline, immediate post participation, and 3 months post participation in the workshop. Thirty-seven parents enrolled in the study and feasibility and acceptability goals were largely met. Parents qualitatively described changes to their parenting, what aspects of the workshop were most helpful, and targeted feedback on how to improve workshop. Findings suggest that providing parents of children with IBD a brief online ACT workshop including nutrition guidance is feasible and leads to changes in parenting behaviours.

## 1. Introduction

The unavoidable physical and emotional discomfort that comes with pediatric inflammatory bowel disease (IBD) can negatively impact all aspects of a young persons’ health-related quality of life (HRQL) [1,2,3,4,5]. Parents are critical partners in their child’s support network and health care team. This is in addition to managing the financial, logistical, emotional, and social demands of parenting in general. Parent mental health and coping play an important role in their child’s adaptation following a diagnosis of IBD [6]. However, parents of children diagnosed with IBD are at increased risk of anxiety, depression, and financial hardship related to the extra demands inherent in their chronically ill child’s care [7].

The impact of a parent’s ability to cope with their child’s IBD can have a direct impact on their child’s ability to cope. Controlling for child age, gender, IBD disease activity, and pain intensity, parent rumination about their child’s pain was uniquely associated with their child’s HRQL. This is over and above the child’s rumination or magnification of their own pain [6]. Moreover, parent behaviours have also been shown to be an important predictor of a child’s report of pain intensity in IBD samples [8,9,10]. These findings highlight the importance of parents’ cognitions and behaviours related to their child’s pain, regardless of their child’s current IBD disease activity. Greater attention to identifying and supporting parents who may struggle with coping is critical for both parent and child overall health. Research has shown that cognitive behavioural and mindfulness-based therapies are equally effective in reducing stress and burn out in parents of children with chronic conditions [11]. However, parents often experience barriers in access to parenting support interventions in pediatric hospital settings (e.g., limited opportunities and trained professionals, access only in large metropolitan locations). Offering online group-based parent acceptance and commitment therapy (ACT) is one solution to provide parents with coping strategies and social support from others with shared lived experience.

ACT is a third wave cognitive behavioural therapy based on six interrelated processes: present moment awareness, acceptance, cognitive defusion, self as context, values, and committed action. The ACT modality emphasizes changing the way one relates to their internal experiences, rather than trying to change the form or content of thoughts and feelings. This approach is ideally suited to individuals with IBD who will inevitably suffer with unwanted physical sensations (e.g., pain, fatigue, and urgency), feelings (e.g., stress, anxiety), and thoughts (e.g., ‘is this the start of a flare-up?’). ACT is a transdiagnostic approach that is efficacious with adults struggling with anxiety and depression and as such, is applicable to parents with a broad range of symptoms and life circumstances [12]. ACT helps individuals develop the skills necessary to flexibly respond to their internal experiences (i.e., thoughts and feelings) and behave in a way that consistent with who and what matters, as opposed to reacting to internal experience in an effort to avoid thoughts and feelings. Empirical findings suggest that ACT is effective for improving HRQL in clinical samples of adults living with chronic illness such as IBD [13,14] and in parents of children with chronic conditions, chronic pain, and anxiety [11,15,16,17,18].

In addition to the need to support coping and increase social support, parents often seek guidance on diet and nutrition in managing their child’s IBD. The use of complementary and alternative medicine (CAM), including nutritional supplements, probiotics, and diets among families affected by IBD is well reported [19,20,21]. This interest and use of CAM is common in pediatric IBD, despite being limited evidence for the role of diet and other types of CAMs in inducing and maintaining disease remission in IBD (excluding exclusive enteral nutrition in pediatric Crohn’s disease) [22,23,24]. Diet was also identified as a top priority research question in a Canadian survey and meeting, highlighting that it is an important topic among families impacted by IBD, and a source of concern and stress [25]. A qualitative study of children with IBD and their families found that most used the identification of specific foods that influence symptoms or trigger flare-ups as a means of coping with IBD [26]. This can lead to food avoidance or exclusion diets in children and adolescents with IBD, which may or may not be necessary and can negatively impact nutritional status [27]. However, this study also found that families emphasized healthy eating in addition to avoidance or moderation of trigger foods, especially due to the difficulty of remaining adherent to exclusion diets (e.g., specific carbohydrate diet) [26]. Similar research among adults with IBD found that adults struggle significantly with decisions about diet, often related to uncertainty of whether a food will exacerbate symptoms or disease, and encounter challenges with abstaining from foods [28]. Patients with IBD in this sample also highlighted the social isolation of adhering to diets, given that food is an integral part of many social events (e.g., birthdays, weddings, and dinner parties) [28].

Taken together, application of evidence-based cognitive behavioural interventions, such as ACT, in combination with knowledge sharing and guidance on nutrition in IBD, is a critical integrated multidisciplinary care approach to support families of children and adolescents with IBD. Offering an ACT workshop online would improve access to supportive care, while decreasing both direct and indirect costs associated with families attending in-person workshops or interventions. The holistic and family-based approach is particularly valuable within pediatric IBD as it can target psychosocial risk factors known to negatively impact health outcomes (e.g., non-adherence, disease activity) [29,30,31]. Therefore, the aims of this work are to develop and test the feasibility, and acceptance and initial effectiveness of a brief group-based online parent workshop, the iACT-P workshop. The iACT-P workshop teaches ACT-based processes, provides guidance in nutrition, and facilitates social support. Our work is guided by the following research questions:

1. What is the acceptability of the iACT-P workshop in terms of recruitment, retention, and perceived parent impact?
2. Do parents attending the iACT-P workshop experience a reduction in stress, anxiety, depression, and increase in psychological flexibility as evidenced by increased cognitive defusion, mindfulness, and values-based living immediately after and 3 months after workshop attendance?

## 2. Materials and Methods

### 2.1. Intervention

The iACT-P workshop included 3 weekly 90 min sessions offered during the evenings on a weekday. Sessions were delivered to parents using Zoom, a secure online video conferencing platform. iACT-P uses concepts of ACT, a third wave cognitive behavioural approach based on six main processes to support psychological flexibility [32]. Parents of children with IBD often express worries about the possibility of a future IBD flare in their child. Instead of working to reframe or dispute these thoughts given the possibility and likelihood of future flares, ACT would teach parents skills to focus awareness and energy on acting based on what is important to them, while taking thoughts and feelings with them, instead of putting time and effort into trying to avoid or get rid of thoughts and feelings. This approach allows parents to develop the skills necessary to respond with flexibility versus react to their internal experiences when parenting while also supporting their child with IBD. The iACT-P workshop series also included structured content and discussions on eating well with IBD, led by a registered dietician (IM) with extensive clinical and research experience working with IBD. All workshop content and discussions were adapted for parents in the pediatric IBD context. iACT-P workshops were facilitated by a licensed clinical and health psychologist (SAK) with a longstanding personal mindfulness practice and advanced training in applied mindfulness, ACT, and teacher training attendance in mindfulness based cognitive therapy. In addition, parents were provided with electronic nutrition in IBD handouts, electronic fillable ACT matrix [33], links to free online guided meditations, apps, and YouTube videos for both parents and children. See Table 1 for session content.

### 2.2. Evaluation

#### 2.2.1. Study Design

A pragmatic prospective prepost study design tested the feasibility and preliminary effectiveness of an online ACT workshop for parents of children with IBD, the iACT-P workshop.

#### 2.2.2. Participants

Study eligibility criteria included inclusion criteria: (a) parents or primary caregiver of a patient attending the IBD program at the Hospital for Sick Children (SickKids) in Toronto, Canada, and (b) ability to speak and read English. Exclusion criteria: (a) significant cognitive or severe psychiatric (e.g., psychosis and active suicidal ideation) impairment in parents thought to impede their ability to participate in the study as determined by their IBD team and (b) no stable internet or data access.

#### 2.2.3. Procedure

The study was conducted according to the guidelines of the Declaration of Helsinki, and approved by the Institutional Review Board of the SickKids Research Institute (#1000063162, 23 July 2019). Following institutional ethics approval, eligible parents of patients currently followed in the IBD program at SickKids were invited to participate using standard recruitment approaches (e.g., letters, posters, and approached during a clinic visit). A member of the IBD team identified eligible parents and introduced them to the study, and if the parent was interested, contact information was shared with a research assistant (RA). The RA obtained consent either in person or via telephone.

Baseline demographic and child disease-related data were obtained from parents at the time of enrolment. All parent outcome measures were administered online using REDCap (research electronic data capture), a secure data collection platform. Participants accessed a unique REDCap survey link via email to complete questionnaire measures at baseline prior to the iACT-P workshop, immediately following the third session, and 3 months following the last session. Workshops were complementary, yet standalone, so parents who did not complete all 3 workshops were still able to complete outcome measures. Parents received a CAD 20 gift card for completing study measures at each timepoint. Following completion of the iACT-P, parents were also invited to complete a program feedback interview on the iACT-P with the purposes of improving feasibility and acceptability in terms of content, structure, and format. Interviews were audio-recorded and the research coordinator took detailed field notes during the interview. Parents were asked about their experience in the iACT-P workshop, any skills gained from the iACT-P workshop, likes and dislikes, and any proposed changes to the structure of the workshop.

#### 2.2.4. Measures

The two main outcomes of this study were acceptability and effectiveness of the iACT-P workshop, as outlined below. All measures are psychometrically sound and have been validated in adult and parent populations, and, when possible, in samples of parents of children with chronic disease. Cronbach alphas were calculated to determine measure internal consistency with 0.70 being deemed acceptable.

Acceptability:

- Recruitment: accrual rate of >70%;
- Retention: attrition rate of <15%, technical difficulties reported by <10% of parents, attendance rate of >80%;
- Parent perceived acceptability of iACT-P: treatment evaluation inventory [34] and qualitative feedback from parents post-iACT-P interview.

Effectiveness:

- Parent reported questionnaires: parent psychological flexibility questionnaire [35,36,37], mindful attention awareness scale [38,39,40], parental acceptance and action questionnaire [41,42], valuing questionnaire [43,44,45], cognitive fusion questionnaire [46,47,48], depression, anxiety, and stress scale [49,50,51], and qualitative feedback from the parent post-iACT-P interview. See Table 2 for description of psychosocial measures.

#### 2.2.5. Data Analysis

Descriptive statistics were used to describe the sample characteristics at baseline using measures of central tendency and dispersion. Rates of accrual, drop out, compliance, and missing data were reported as percentages. Effectiveness outcome data will be analyzed using an intent to treat approach. Repeated measures analysis of variance was conducted on all quantitative parent reported outcomes. In addition, individual meaningful differences were calculated for each parent and reported in a posthoc analysis. We used a measure of 0.5 of standard deviation to define meaningful change on a measure. This was chosen based on the literature as the most conservative estimate for our purposes of meaningful improvement [52,53]. This patient centered approach to outcome reporting was explored due to the various needs and preferences reported by parents (e.g., attending group for social support vs. for mindfulness vs. for guidance on nutrition vs. prevention). Lastly, all feedback interviews were transcribed verbatim and interview audio files were reviewed for fidelity. Qualitative data were then analyzed using simple content analysis with coding to consensus and reviewed by 3 investigators with experience in qualitative data analysis.

## 3. Results

### 3.1. Study Participants

Thirty-seven parents were enrolled into the study and 32 participated in an iACT-P workshop between November 2019 to July 2020 (see Table 3). Informed consent was obtained from all subjects involved in the study. All were biological parents of a child with IBD, primarily mothers of various ethnic backgrounds, with most graduating college or university. Of the 37, 23 parents completed a qualitative interview post iACT-P workshop (interviews conducted between December 2019 and August 2020).

### 3.2. Treatment Acceptability

#### 3.2.1. Recruitment

A total of 72 parents were screened and offered the iACT-P workshop. Of those, 37 were enrolled (51.4%) and an additional six were on a waitlist for the next group at the time of study completion due to scheduling. Of the 29 who did not enrol, 15 (20.8%) were not interested and 14 (19.4%) were interested but lost to follow-up. Initial recruitment goals of >70% were not met.

#### 3.2.2. Retention

Of the 37 parents enrolled in iACT-P workshop, three parents withdrew (8%), one prior to attending any sessions and two after attending one session. No parents declined participation due to technological issues nor did any parent experience technological issues that did not resolve within 10 min of the initial session. Attendance across groups was 86.5% overall, with 68.75% of parents attending all three sessions, 21.88% attending two sessions, and the remaining 9.37% attending one session. With respect to sessions, 90.63% of parents attended the first session and 78.13% attended both the first and second session. All initial retention goals were met.

#### 3.2.3. Parent Perceived Acceptability

All parents in attendance participated throughout the sessions and in all activities. Although parents at times needed to excuse themselves, none left the video screen for longer than 10 min. Responses on the treatment evaluation inventory found overall acceptability of the iACT-P workshop for parents with an overall average of agree (mean = 4.00, standard deviation = 0.79) with all items. Parents endorsed agree and strongly agree with the following statements: 62.5% found iACT-P an acceptable way to deal with child’s IBD (28% felt neutral), 81% would be willing to use this approach to change child’s health behaviours, 73% liked the approaches used, 82% believe the iACT-P workshop is effective, 72% believe the iACT-P workshop is likely to result in permanent improvement, and 88% had a positive reaction to the iACT-P workshop. During qualitative feedback on the length of the workshop, 74% of parents reported that 3 weeks was a sufficient length while 26% reported this amount of time was too short. When asked, 70% of parents would have been open to more weeks in the intervention program. With respect to the length of individual session, 61% reported that 90-min sessions were the right length while the remining 39% reported that session length was too long. For format of the sessions, 83% of participants reported that participating in the program through online videoconference was suitable while 17% would have still preferred to participate in-person. All parents would recommend the iACT-P workshop to other parents of children with IBD. Most common reasons for recommending the iACT-P workshop include: sharing ideas and talking to other parents, providing parents support, especially if they are isolated, provides parents strategies (i.e., mindfulness and meditations) to deal with stress, the group helps with anxiety, provides tools to help with parenting more broadly and helps parents to know they are not alone in parenting a child with IBD.

Parents also provided feedback on how they would like to be supported more broadly. Firstly, parents overwhelmingly requested more guidance and information on food and access to dedicated IBD nutrition support, e.g., an IBD dietitian. Parents described the IBD clinic as being focused on medication and excluded conversation about diet, naturopathic, or alternative approaches to managing IBD. In particular, parents would like more guidance on the role of food in IBD and how parents can support their child’s diet and nutritional status. Parents also suggested that the hospital provide more opportunities for parents to have social gatherings. Suggestions for formats included an online group or in person coffee nights to provide a platform for parents to interact once a month to once every few months in order to exchange ideas, information, and offer peer support and shared experiences. Finally, parents requested more information on healthy living (nutrition, exercise, and mental health) as it pertains to children and IBD. All acceptability goals were met, however adaptations to be considered based on parent feedback are included in Table 4.

### 3.3. Preliminary Effectiveness

#### 3.3.1. Quantitative Outcomes

There were no significant group improvements in any quantitatively measured domains following participating in the iACT-P workshop (see Table 5). However, when exploring individual meaningful differences (defined as a change >0.5 standard deviations (SD) observed in the sample at baseline), it was noted that six parents (21.4%) did not report any improvements, while nine parents reported improvements in psychological flexibility (parent psychological flexibility questionnaire-PPFQ) and an additional 13 reported improvements in a minimum of one ACT process (mindful attention awareness scale-MAAS, parental acceptance and action questionnaire-PAAQ, cognitive fusion questionnaire-CFQ, and valuing questionnaire-VQ). Thus, 78.6% of parents reported individual meaningful improvements in overall psychological flexibility or core ACT processes after participating in the iACT-P group. With respects to symptoms of anxiety, 20 (69.0%) parents were within normal limits at baseline and of the remaining 9 parents, 4 (13.8%) parents improved their classifications post iACT-P group attendance while 5 did not change. Similarly, 19 (65.5%) parents were within normal limits at baseline for symptoms of stress, of the remaining 10 parents, 5 (50.0%) improved their classifications post iACT-P workshop. Lastly, 20 (69.0%) parents were within normal range for symptoms of depression at baseline, with 2 (22.2%) of the remaining 9 parents improving a classification post iACT-P.

#### 3.3.2. Qualitative Outcomes

The content analysis revealed two main categories with subcategories that contribute to parents’ qualitative outcomes post iACT-P workshop participation. Parents discussed what they found most helpful about the iACT-P workshop and how they have changed as parents after participating in the program. What was most helpful was the opportunity for connection over shared lived experience, connecting to allied health, and learning mindfulness. How they have changed as parents included increased awareness, the ability to stay calm, and letting go of the need to ‘fix’ everything.

**What was most helpful about the iACT-P program**: Parents reported what they found most helpful about the iACT-P workshop. Overwhelmingly, the first theme parents spoke to was how the group allowed them to see that they were not alone in their journey parenting a child with IBD. This sharing of lived experiences with other parents was a comfort and was repeatedly shared in feedback interviews as being the most helpful aspect of participating in the group. As one parent explains:

*What I realized, I know there are other parents too, but now I realize lots of parents with kids they are just like us and they are also having the same problems we are having so it was good when we met a group of parents and when they tell us their stories, we feel more confident, more brave. there are other people too facing these kinds of issues and this made me feel that we aren’t alone. P011*

The sentiment of not feeling alone and feeling supported by other parents was also described by some as being a part of a community where members can relate to one another. This parent also speaks to the normalizing effect of the group:

*I liked that there were other parents involved because then it makes you realize you aren’t an island and you aren’t the only one going through it and other families are experiencing the same things with a child with IBD and realizing you aren’t the only one and giving you a place to see how other people are handling it. P017*

Other parents spoke about feeling validated as the biggest takeaway from the group. The recognition by others, and in particular the IBD psychologist, about the challenges of parenting children with IBD was explained as a helpful outcome of the group. One parent explains being validated as follows:

*…hearing [facilitator] talk about validating for us as parents how hard it is and honestly if that’s all I heard in my 3 weeks that was the greatest thing I could have heard because we aren’t hearing that in terms of when we go to clinic it is all about the child as it should be and so we aren’t getting that support, we get it from each other I’ve been active in trying to create parent groups so we are finding those ways to get some of that validation with other parents but to hear the psychologist say that validated every heartache and frustration that I feel. P008*

A second theme centered around facilitating connections to allied health care professionals, in particular the dietitian with experience in IBD and health psychologist. Having the dietitian attend the iACT-P workshop was cited by the majority of parents as being one of the most helpful aspects of the group. Parents wanted additional time and guidance from the dietitian. One parent noted that in her group, parents were asking the dietitian very basic or introductory questions. She explains:

*…it also tells me that we are still not feeling as informed as we would like to be. In 6 years, I’ve never been handed a piece of paper and I’m very much ‘knowledge is power’. As parents there is so much frustration because we can’t take away what our kids are going through. But, if we are more informed, and the better informed we are, I think we feel better in the process and more empowered in the process. P008*

A third theme was how helpful the mindfulness and meditation techniques were in helping parents to manage the stress of parenting a child with chronic illness. Perspective taking tools that facilitated present moment awareness helped parents reflect on whether they were acting according to their values (vs. avoidance behaviours). Using tools such as the matrix were also discussed as strategies parents continued to use after the group in order to evaluate priorities in day-to-day life. As one parent explains, even though meditation was not a practice she would typically enjoy, she still found mindfulness to be meaningful and helpful in her parenting role:

*…mindfulness, it’s honestly it’s not my thing and my 15 year old with Crohn’s its not her thing and we are trying to embrace it a little more because there were some really good tips but I’ve come on board a little more. P019*

**How you have changed as a parent after the iACT-P program**: Parents reflected upon changes they saw in themselves as a result of being in the iACT-P workshop across three themes. The way in which parents support their child through the challenges of IBD was one notable change. In particular, parents explained that they now allow space for negative experiences and do not always strive to “fix” problems their children experience. This parent comments:

*The only thing that really hit home was one specific point which was…the point was made about how sometimes kids come to you and they sort of dump their problems on you, but they don’t really want you to solve their problems. They don’t want you to give them solutions they just want you to listen and as parents we tend to want to fix things for them and sometimes that’s not always what we should be doing or that’s maybe not what they want us to do. And that was probably the most significant point that I took home from the 3 sessions. P023*

A second parent shares these sentiments:

*One of the biggest skills I learned from this was lay off. Lay off your child. Let him be. Let him breathe. Let him figure it out. And he voices that all the time. But I always thought, I’m a mom, he’s a teenager, let him keep yelling at me and saying, ‘mom stop this, stop this, why do you always keep asking me?’ I thought I just always have to continue being persistent because that’s who we are, we’re parents, so you keep pushing forward. But when a child is dealing with an illness, I think as a parent you just need to give them time to breathe and let your child come to you. And I think that is my biggest advantage or the biggest learning piece I’ve taken away from this. P009*

Increased awareness in everyday life was a second change reported by parents. Parents reported an increase in their ability to focus on one thing at a time or to take a moment to enjoy what they were doing following their participation in the iACT-P workshop. By taking a step back and rethinking one’s approach to their child’s IBD, parents discuss how the function of their actions may be categorized as either moving away from thoughts and feelings, or towards their values and goals with respect to their relationship with their child.

*The whole mindfulness piece really comes across loud and clear. I never thought of it in relation to my child it was always in my own day to day life but thinking about how to manage that kind of made me more aware and now taking a bit of a step back with what’s happening and especially because we’ve been going through a pretty rough time in the past couple of weeks. P027*

The improved ability to work with difficult situations and invite a ‘calm’ defused approach as a means of self-care was a third change. Parents described that if one is able to work through difficult internal experiences by taking care of one’s self, this will have a trickle-down effect to the children as they witness both self-care and responding (instead of reacting). Although ‘staying calm’ is not aligned with the ACT processes, during interviews parents described working with and accepting unwanted internal experiences as being able to be ‘more calm’. The following parent explains this as her takeaway from the program:

*…trying to stay calm through whatever experience or difficult time you are going through and take care of yourself, learn to take care of yourself because if you take care of yourself you can take care of your children. P031*

## 4. Discussion

This study sought to explore the feasibility, acceptability, and initial effectiveness of an online ACT and nutrition workshop, the iACT-P workshop, for parents of children with IBD. While initial recruitment fell slightly short of a priori goals (goal of >70%, actual recruitment 51.4%), it parallels the literature on interventions targeting parents of chronically ill children or children with intellectual delays [54,55,56]. All other measures of feasibility and acceptability were met. Several adaptations were suggested by parents to further support implementation as a part of routine integrated multidisciplinary clinical care, namely the shortening of sessions and the addition of a 4th session. This will allow for content and main discussion to end shortly after an hour and leave space for continued discussion for those interested. Consideration for ongoing monthly drop-in parent support sessions should also be explored.

Surprisingly, there were no significant improvements in the overall analysis of parent reported outcomes. The literature in online ACT interventions for parents often report significant improvements in parent stress, depression, and anxiety, although symptom reduction is not a goal of ACT [17,56,57,58,59]. In exploring the individual differences in parents, the majority reported improvements in either psychological flexibility or some underlying ACT processes. Improvements in ACT processes (e.g., mindfulness, acceptance, valued living, and cognitive defusion) but not parent stress, anxiety, or depression, suggest that parents’ ability to work with symptoms improved during the study, and while symptoms still present, they were not as disruptive to their lives. This is clinically meaningful as some degree of stress, anxiety, or depression while parenting a chronically ill child is to be expected and therefore important to equip parents with skills to work with these experiences. Indeed, research has suggested that ACT may be ideally suited to be provided in an acute pediatric hospital for this reason [17]. Alternatively, the lack of significant findings may be, in part, due to the differences in parent needs prior to attending the iACT workshop. The workshop was intended to be a component of integrated multidisciplinary care and as such as inclusive as possible; allowing for parents to enrol for prevention or building resilience vs. a focus on parents who are experiencing psychological distress (or have children experiencing psychological distress). As a result, our sample reported mean symptoms of stress and anxiety in the mild range and depression in the normal range at baseline. Future research with larger samples may be able to identify whether there is a parent phenotype that would benefit most from this type of workshop and thereby can be targeted, ideally early in their child’s journey with IBD in order to prevent worsening stress or anxiety. Still, the group discussions were enriched by having a myriad of experiences with respect to parent’s own mental health, their child’s mental health, and their child’s IBD disease severity. Parents appreciated the ability to see the range of experiences of pediatric IBD, both in terms of disease presentation but also implications at different developmental ages. Parents qualitative reports are promising with respects to changes in their parenting behaviours and being indicative of an incorporation of mindfulness into everyday life. Their descriptions represented an increase in awareness, such as responding to their child instead of reacting.

A strong theme across all sessions and outcomes were parents’ strong desire to have a forum for sharing lived experiences of parenting a child with IBD. Our findings are similar to those in the literature whereby systematic reviews of parent interventions in chronic disease find consistently strong positive qualitative findings but inconsistent results on parent reported quantitative outcomes [60]. Our findings would support the need for ongoing parent social support paired with education on living well with IBD, including a holistic approach to care. For example, research in adults with IBD has highlighted the nuanced importance of acceptance vs. tolerance of IBD and its symptoms [61]. ACT skills paired with social support approaches would be particularly helpful in this regard as acceptance is a core ACT process and our findings demonstrate shared lived experience normalizes the illness experience.

Our findings also underscore the need for guidance in diet and nutrition within pediatric IBD clinics. Studies of parents of children with IBD have found that many believe that food is a cause of IBD and 61.5% avoid certain foods in fear of exacerbating symptoms for their children [62]. In adults with IBD, a lack of knowledge of whether dietary changes (e.g., food avoidance and exclusion diets) impacted IBD symptoms paired with those dietary changes lead to maladaptive coping and social isolation that in turn decreased psychosocial functioning [63]. In Canada, both pediatric and adult IBD experience ongoing barriers to accessing more holistic care, including psychosocial and nutrition care [64,65]. Internationally, access to ‘high quality written’ IBD-specific nutrition information has also been found to be variable [66]. Our findings alongside the literature demonstrate that there is a need to explore ways in which to increase access to both psychosocial and nutrition in IBD over the lifespan.

### Limitations

Findings from this pilot study should be interpreted in light of several considerations. The recruitment and data collection for this study occurred shortly prior to and during the coronavirus disease 2019 (COVID-19) pandemic. Parents in different cohorts of the iACT-P workshop would have been in different stages of COVID-19 restrictions during both outcome measurement and workshop attendance. Although COVID-19 was a part of conversations, the content of the iACT-P workshop remained primarily focused on parenting in IBD and how COVID-19 has impacted the child relative to IBD. Given the focus on feasibility for this pilot study, our sample size was small and there was no control group data collected. Further research is warranted to determine whether parent reported qualitative improvements are directly the result of the iACT-P workshop.

## 5. Conclusions

Parenting in the context of pediatric IBD necessitates navigation of medical appointments, procedures, and medications in addition to other parent responsibilities. As essential members of a child’s healthcare team, it is crucial to support parents in caring for their child with IBD. Family based approaches that target parents and include both psychosocial and nutritional components are an example of providing integrated multidisciplinary care in pediatric IBD clinics. Our findings suggest that a brief online parent workshop, the iACT-P workshop is a feasible and acceptable approach to supporting parents in the pediatric IBD context. Although no significant improvements were seen via questionnaires, parents qualitatively shared that they benefited from sharing lived experiences, increased awareness and integrated mindfulness, and more clarity on eating well for IBD. Furthermore, results support the need and preference of families for integrated dietician and mental health supports for IBD clinical care. Future research into the effectiveness of the iACT-P and the impact of incorporating additional ongoing maintenance supports (e.g., monthly drop-in sessions) is warranted.

## Figures and Tables

**Table 1:** iACT-P workshop session content.

Session Number	Concepts Covered	Exercises and Activities
1	Psychoeducation on parenting in IBD context (including developmental considerations for growing up with IBD) Introduction to Mindfulness Using values to guide parenting choices and behaviours	Group sharing of brief IBD journey Focused attention and grounding practice Group ACT Matrix Group discussion
2	Eating well for IBD Integrated/applied mindfulness Gratitude Noticing your towards and away moves	Mindful Eating Joy and savoring practice STOP practice ACT Matrix Group discussion
3	Noticing your own and your child’s towards and away moves Compassion for self and others	Awareness, gather, and expand practice Self-compassion break Loving Kindness practice Group discussion Committed Action

Note: IBD—inflammatory bowel disease, ACT—acceptance and commitment therapy, STOP—stop, take a breath, observe, proceed.

**Table 2:** Psychosocial measures assessed at baseline, immediately postintervention, and 3-months post-intervention.

Measure	Domain Measured	Subscales	Score Range	Cronbach’s Alpha (Baseline, Immediate, 3-Month)
Parent Psychological Flexibility Questionnaire [35] (PPFQ, 17 items)	Psychological flexibility	Values-based action, emotional acceptance, pain acceptance, pain willingness	Total score: 0–102 Higher scores indicate greater flexibility	0.93
				0.95
				0.96
Mindful Attention Awareness Scale [38] (MAAS, 15 items)	Mindfulness	-	Average score: 1–6 Higher scores indicate greater mindfulness	0.92
				0.94
				0.94
Parental Acceptance and Action Questionnaire [41] (PAAQ, 15 items)	Experiential avoidance	Inaction, unwillingness	Total score: 0–105 Lower scores indicate a lower level of parental experiential avoidance	0.72
				0.70
				0.74
Valuing Questionnaire [43] (VQ, 10 items)	Valued living	Progress, obstruction	Total score per subscale: 0–30 Higher progress scores reflect greater enactment of values Lower obstruction scores reflect lower disruption of valued living	Progress:
				0.89
				0.83
				0.87
				Obstruction:
				0.87
				0.81
				0.86
Cognitive Fusion Questionnaire [46] (CFQ, 7 items)	Cognitive fusion	-	Total score: 7–49Lower scores indicate a lower level of cognitive fusion	0.97
				0.96
				0.96
Depression, Anxiety and Stress Scale [49] (DASS, 21 items)	Symptoms of depression, anxiety, and stress	Depression, anxiety, stress	Total score per subscale: 0–42Lower scores indicate fewer symptoms of depression, anxiety, and stress Scores are classified as normal, mild, moderate, severe, and extremely severe	Depression:
				0.93
				0.91
				0.90
				Anxiety:
				0.91
				0.80
				0.82
				Stress:
				0.84
				0.85
				0.89

**Table 3:** Parent Demographics (*n* = 37).

**Age**	
30–39 years old *n (%)*	8 (22%)
40–49 years old *n (%)*	22 (59%)
50–59 years old *n (%)*	7 (19%)
**Sex**	
Male *n (%)*	5 (14%)
Female *n (%)*	32 (86%)
**Self-Identified Ethnicity**	
North American *n (%)*	10 (27%)
South American *n (%)*	1 (3%)
Caribbean *n (%)*	1 (3%)
European *n (%)*	6 (16%)
Jewish *n (%)*	4 (11%)
Middle Eastern *n (%)*	4 (11%)
East Asian *n (%)*	1 (3%)
South or Southeast Asian *n (%)*	10 (27%)
**Education**	
Some college/technical school *n (%)*	2 (5%)
Graduated college/technical school *n (%)*	26 (70%)
Graduate degree *n (%)*	9 (25%)
**Employment**	
Unemployed *n (%)*	4 (11%)
Part-time *n (%)*	6 (16%)
Full-time *n (%)*	26 (70%)
Retired *n (%)*	1 (3%)
**Income**	
Less than $25,000 *n (%)*	2 (5%)
$25,000 to $49,999 *n (%)*	0 (0%)
$50,000 to $74,999 *n (%)*	4 (11%)
$75,000 to $99,999 *n (%)*	4 (11%)
$100,000 to $150,000 *n (%)*	7 (19%)
Above $150,000 *n (%)*	13 (35%)
Did not wish to answer *n (%)*	7 (19%)
**Marital Status**	
Married or living common-law *n (%)*	33 (89%)
Widow or widower *n (%)*	1 (3%)
Separated *n (%)*	1 (3%)
Divorced *n (%)*	2 (5%)
**Child’s Demographics**	
Age, years *mean (SD*)	12.27 (3.91)
Age, years *range*	4–17
**Sex**	
Female *n (%)*	21 (57%)
Male *n (%)*	16 (43%)
**Diagnosis**	
Crohn’s *n (%)*	19 (51%)
Ulcerative Colitis *n (%)*	16 (43%)
IBD-U *n (%)*	2 (6%)
Years since child’s diagnosis, years *mean (SD*)	2.14 (2.23)
Years since child’s diagnosis, years *range*	0–6

Note: SD—standard deviation, IBD-U—inflammatory bowel disease unclassified.

**Table 4:** Suggested adaptations to the iACT-P workshop.

Parent Feedback	iACT-P Adaptation
Sessions of 90 min were too long	Shorten sessions to 60–75 min
Request for more in-depth nutrition guidance	Add a 4th session to ensure additional time with a dietician for expanded content and additional discussion at the end
Request for more discussion and space to ask each other questions	End session content by 60 min and allow for additional discussion at the end for parents who are interested Additional 4th session allows extra time for discussion during and after session content

**Table 5:** Preliminary outcome measures.

Measure	Mean (SD)								
	Range	*N*	Pre	*N*	Post	*N*	3-Month	*F*	*p*
PPFQ—Total Score	0–102	34	45.34 (17.37)	31	44.88 (21.16)	30	47.21 (21.68)	1.38	0.26
MAAS—Total Score	1–6	35	3.96 (0.90)	33	3.66 (0.89)	33	4.02 (0.93)	0.12	0.89
PAAQ—Total Score	15–105	34	64.50 (10.46)	35	63.80 (9.99)	29	63.21 (9.74)	0.53	0.60
CFQ–Total Score	7–49	34	23.12 (10.37)	32	23.00 (9.56)	30	21.09 (10.22)	2.89	0.06
**VQ**									
Progress	0–30	35	20.52 (5.57)	32	19.70 (5.48)	29	19.45 (6.63)	0.30	0.74
Obstruction	0–30	34	10.97 (6.56)	32	11.44 (5.80)	29	10.45 (6.49)	0.09	0.91
**DASS**									
Depression	0–42	34	8.59 (8.38)	32	9.50 (7.48)	28	8.35 (7.30)	0.20	0.82
Anxiety	0–42	34	7.90 (8.78)	32	9.50 (7.80)	28	8.57 (6.93)	0.25	0.78
Stress	0–42	34	15.51 (7.64)	32	15.39 (7.63)	28	13.54 (7.74)	0.27	0.76

Notes: SD—standard deviation, PPFQ—parent psychological flexibility questionnaire, MAAS—mindful attention awareness scale, PAAQ—parental acceptance and action questionnaire, VQ—valuing questionnaire, CFQ—cognitive fusion questionnaire, DASS—depression, anxiety, and stress scale.

## Data Availability

The raw data supporting the conclusions of this article will be made available by the authors, without undue reservation to qualified researchers.
